# Corneal nerve fiber size adds utility to the diagnosis and assessment of therapeutic response in patients with small fiber neuropathy

**DOI:** 10.1038/s41598-018-23107-w

**Published:** 2018-03-16

**Authors:** Michael Brines, Daniel A. Culver, Maryam Ferdousi, Martijn R. Tannemaat, Monique van Velzen, Albert Dahan, Rayaz A. Malik

**Affiliations:** 1 Araim Pharmaceuticals, Tarrytown, NY USA; 2 Pulmonary Medicine, Cleveland Clinic, Cleveland, OH USA; 3 Divison of Cardiovascular Sciences, University of Manchester, Manchester, United Kingdom; 4 Neurology, Leiden University Medical Center, Leiden, The Netherlands; 5 Anesthesiology, Leiden University Medical Center, Leiden, The Netherlands; 6 Weill Cornell Medicine-Qatar, Doha, Qatar

## Abstract

Small fiber neuropathy (SFN) is a common feature of many inflammatory diseases, often presenting with pain and disability. SFN is diagnosed using symptoms, thermal threshold testing, and intra-epidermal nerve fiber quantification. Corneal confocal microscopy (CCM) is an ophthalmic imaging technique which non-invasively quantifies corneal nerve fiber (CNF) density, branch density and length, and has comparable diagnostic and superior ability to identify nerve regeneration compared to skin biopsy. CNF size (width and area) depends upon the number of fibers within each nerve, as well as pathology (e.g., swelling), and may provide additional sensitivity to diagnose SFN and identify nerve repair. We have compared the utility of the standard CCM variables employed to CNF size in patients with diabetic sensorimotor polyneuropathy or sarcoidosis-associated SFN, and in patients with SFN following cibinetide administration, an agent which promotes nerve repair. The results show that: 1) CNF width distribution and area depend upon neuropathy severity; 2) CNF area, density, branch density and length possess comparable discriminatory power for diagnosing neuropathy; 3) CNF area is related to length by a quadratic function which is predictive for both healthy subjects and those with SFN; 4) CNF area is a useful variable for quantifying change in CNF morphology.

## Introduction

Small Aδ and C nerve fibers of the sensory and autonomic nervous systems constitute 70–90% of peripheral nerve fibers^1^. Peripheral neuropathies, particularly those associated with chronic inflammation, e.g., diabetes^2^, are characterized by a preferential decrease in these small fibers^3^, often occurring early in the course of disease. This small nerve fiber loss (SNFL) commonly presents with pain, dysesthesia, and/or autonomic dysfunction^4^. The development of reliable, objective methods to diagnose and follow the clinical course of SNFL has become a priority in the neurological and pain medicine fields^5^.

To date, the objective assessment of SNFL has primarily relied on the quantification of intra-epidermal nerve fiber density (IENFD) in skin biopsies^6^. However, this procedure is invasive, cannot be truly replicated, is time consuming, and requires expert histological technique and analysis to provide reliable data. Over the last decade, quantification of small nerve fiber abundance in the cornea using *in vivo* corneal confocal microscopy (CCM) has been increasingly used for patients with diabetic polyneuropathy^7^ hereditary neuropathy^8^, Human Immunodeficiency Virus- associated sensory neuropathy^9^, chemotherapy-induced neuropathy^10^, as well as amyloid^11^ and chronic inflammatory demyelinating polyneuropathy^12^. Indeed, several studies have confirmed that CCM has a comparable sensitivity and specificity to IENFD in the diagnosis of diabetic neuropathy^13,14^. Major advantages of CCM, as compared to skin biopsies, are that it is quick, non-invasive, and allows multiple replicates in both cross-sectional and longitudinal studies. Corneal nerve quantification has been accomplished using manual, semi-automated, or fully automated techniques^15,16^.

**Figure 1:**
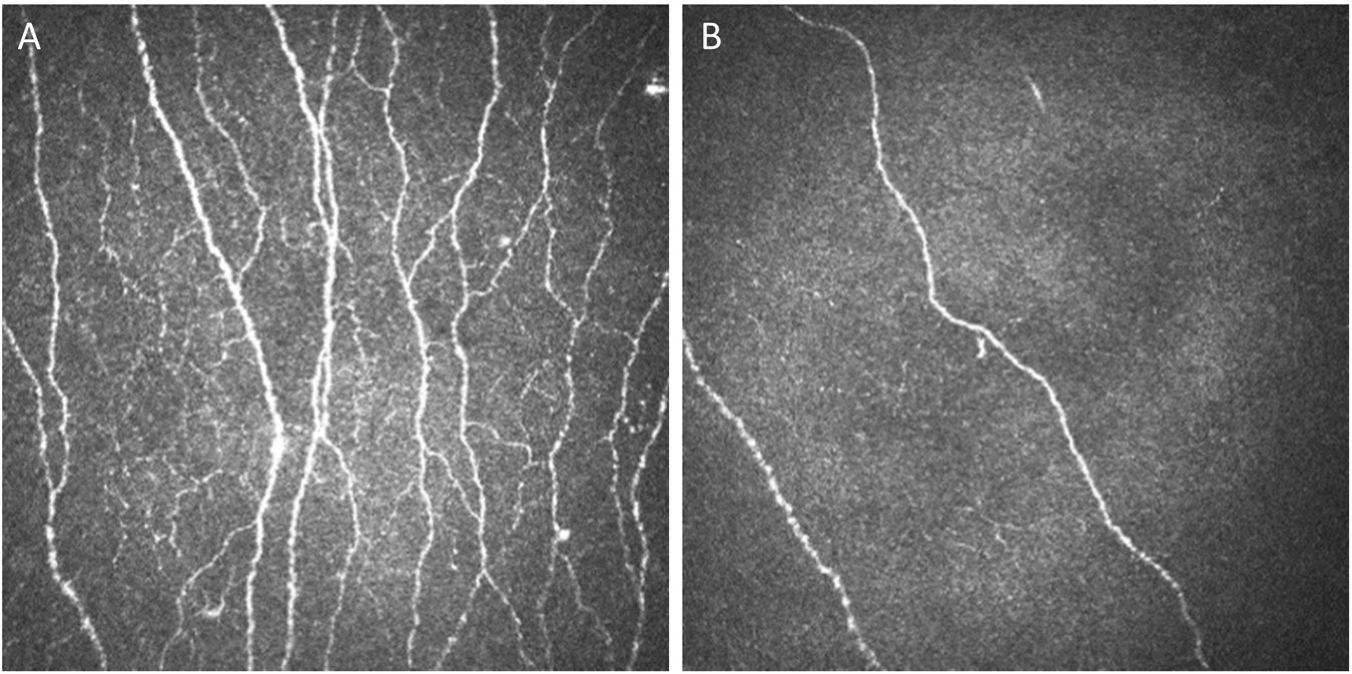
(**A**) The corneal subasal epithelial nerve plexus is normally highly arborated with fiber bundles of varying caliber (normal subject). (**B**) In contrast, subjects having small fiber neuropathy exhibit a reduction in the number of nerve fiber bundles and loss of complexity, including variation in nerve bundle size (subject with severe diabetic neuropathy).

The morphology of corneal nerves imaged by the CCM technique is complex (Fig. 1). Innervation of the cornea is comprised almost entirely of unmyelinated C-type sensory fibers^17^ which are very small, ranging in width from 0.2–2.0 µm. Ultrastructural studies using electron and light microscopy^18^ have confirmed that individual nerve fibers are often below the resolution of CCM^19^, such that most of the wider linear structures visualized in the sub-basal nerve layer using confocal microscopy represent nerve bundles containing groups of individual nerve fibers (up to 30 in normal individuals)^20^. The width (caliber) of these nerve fiber bundles varies in proportion to the number of fibers contained within. Additionally, main nerve fiber bundles branch to a variable degree, exhibit beading, corresponding to regions of mitochondrial accumulation^18^, and exhibit swelling in neuropathy^21^. Therefore, in SFN the total number and width of the main nerve fiber bundles, branching and length^15^, and the spacing and size of the beads^22^ change in complex ways as a function of neuropathy severity.

To date, the evaluation of corneal nerve fibers (CNF) has relied primarily on one dimensional variables for quantifying the number of fibers (nerve fiber density; NFD), number of branches from the principal fibers (nerve branch density; NBD), and nerve fiber length (NFL). It has been suggested, based on reproducibility^23^ and concurrent validity with the clinical and electrophysiological examinations^24^, that the single best assessment of CNF quantification is CNFL. However, the full severity of neuropathy may not be captured utilizing variables that are insensitive to changes in fiber size, i.e., the number of bundled individual nerve fibers, or beading size and frequency. Corneal nerve fiber area (NFA) i.e., the spatial extent of each nerve fiber bundle, is a two-dimensional metric that could provide additional information and more fully capture the full spectrum of variation of the corneal nerve plexus.

The purpose of the present study was three-fold. First, to compare using automated methodology the sensitivity and specificity of NFD, NBD, NFL, and NFA for discriminating subjects having neuropathy from normal controls. To accomplish this, two different methods for calculating NFA were used: 1) the width of the nerve fiber bundle multiplied by its length NFA WxL) as determined by a widely used automated image analysis program (ACCmetrics) and 2) quantifying the total number of pixels contained within the corneal nerve plexus of the CCM image (NFA FIJI). The second goal was to compare CNF width and area in patients with diabetes- and sarcoidosis-associated neuropathy to determine whether differences exist between the two groups. The third aim was to determine the sensitivity of each CCM variable to detect nerve fiber changes following short-term administration of cibinetide, an engineered innate repair receptor ligand with neuroprotective and neurorestorative properties^25^ in patients with sarcoid-associated SNF loss^26–28^.

## Results

### Comparison of CCM measurements obtained from control subjects versus those having diabetes or sarcoidosis

**Figure 2:**
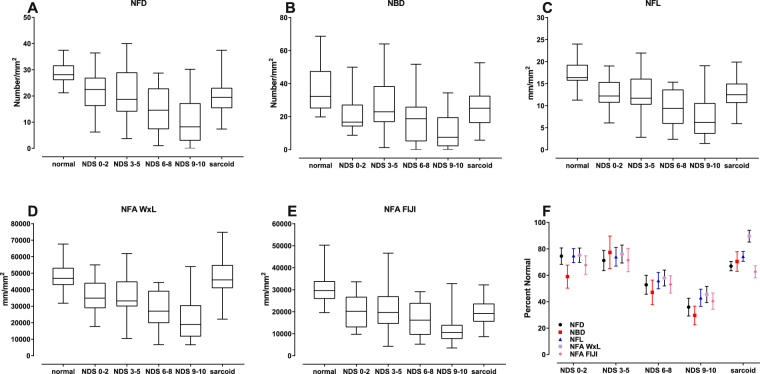
Descriptive statistics of corneal confocal microscopy (CCM) variables (**A–E**) (median with interquartile range and maximum/minimum). In general, values of all variables decrease with increasing severity of neuropathy. The sarcoidosis group was generally similar to the NDS (0–2) and (3–5) groups for NFD, NBD, NFL, NFA FIJI, but diverged for NFA WxL. (**F**) Percent reduction from the corresponding means of normal subjects for each CCM variable (error bars: SEM) illustrates that small nerve fiber losses range from 80% to 40% with increasing severity of neuropathy. [NFD = nerve fiber density; NBD = main nerve branch density; NFL = nerve fiber length; NFA WxL = nerve fiber area determined by length times average width; NFA FIJI = nerve fiber area determined by the the total number of pixels within the nerve plexus].

The demographics of the groups studied are summarized in Table 1. Although the data spread was relatively wide for each CCM variable, the median values obtained using both automated methods generally exhibited a progressive reduction in value with increasing Neuropathy Disability Score (NDS; Figure 2; Table 2). However, subjects without neuropathy (NDS 0–2) deviated from what is otherwise a negative monotonic curve, especially for NBD, such that this group was similar to the NDS 3–5 group. Adjustment for the significantly younger mean age (a weak determinant of corneal nerve fiber abundance^29^) of the NDS 0–2 group using a linear model with age as a covariate did not affect this deviation (data not shown). The measurements obtained from the sarcoidosis group were also very similar to the NDS 3–5 group for most CCM variables, except for NFA WxL (Fig. 2D), which surprisingly was similar to the distribution of the normal subjects. When expressed as percent of the mean value of control subjects (Fig. 2F), no significant differences were noted between the neuropathy groups, except for NFA WxL in the sarcoidosis group which was significantly different from NFD (p = 0.005), NBD (p = 0.026), and NFA FIJI (p < 0.001). Further, the relative ranks of the means of each CCM variable expressed as percent of the control group mean were generally maintained across the groups, except for NFD and NFA WxL in the diabetic patients with NDS 0–2 and sarcoidosis subjects, respectively.

**Table 1:** Demographics of subject populations.

group	n	males	age	Disease duration (years)	BMI	HbA1c (%)	NDS
Diabetes^a^ NDS (0–2)	21	15	37.1 (16.5)^b,c^	17.9 (15.1)	26.4 (5.5)	7.9 (1.3)	0.381 (0.740)
Diabetes^a^ NDS (3–5)	21	12	55.9 (11.0)	33.5 (16.7)	28.9 (3.8)	8.6 (1.4)	3.810 (0.928)
Diabetes^a^ NDS (6–8)	19	11	59.0 (11.3)	44.3 (11.0)	28.4 (3.8)	8.0 (1.1)	6.895 (0.875)
Diabetes^a^ NDS (9–10)	20	12	57.0 (14.6)	28.1 (15.1)	24.9 (7.2)	7.8 (2.1)	9.800 (0.410)
Control	48	25	46.2 (16.9)	NA	26.4 (5.5)	5.7 (0.3)	0.425 (1.231)
Sarcoidosis	63	31	50.2 (10.0)	11.1 (9.3)	29.3 (5.5)	ND	ND
Type 2 Diabetes^d^	48	30	63.1 (7.0)	ND	30.7 (4.4)	7.2 (1.4)	ND

^a^Diabetes group 1.

^b^Significantly younger than the other groups.

^c^Mean (standard deviation).

^d^Diabetes group 2.

NA: not applicable; ND: not determined.

**Table 2:** Corneal nerve fiber analysis variables.

group	NFD number/mm^2^	NBD number/mm^2^	NFL mm/mm^2^	NFA WxL µm/mm^2^	NFA FIJI µm/mm^2^
Diabetes NDS (0–2)	21.5 (7.3)^a^ 6.3–36.5 (22.5)^b^	21.6 (11.7) 8.8–50.0 (16.7)	12.8 (3.4) 6.1–19.0 (12.2)	39385 (10626) 19297–59771 (37461)	20966 (7718) 9792–33607 (20176)
Diabetes NDS (3–5)	20.5 (9.5) 3.1–40. (18.8)	28.2 (17.3) 1.0–64.1 (22.9)	12.7 (5.0) 2.4–22.0 (11.7)	39843 (14474) 11383–67150 (36511)	22122 (10740) 4333–46624 (19694)
Diabetes NDS (6–8)	15.2 (8.7) 1.0–28.8 (14.6)	17.2 (13.2) 0–51.8 (18.8)	9.6 (4.3) 2.4–15.3 (9.4)	30340 (12571) 7274–48140 (29335)	16414 (7594) 5234–29111 (16196)
Diabetes NDS (9–10)	10.3 (8.5) 0–30.2 (8.2)	10.8 (10.7) 0–34.4 (7.5)	7.3 (4.7) 1.4–19.0 (6.2)	28827 (13603) 7217–58661 (20523)	12516 (7773) 3563–32794 (10573)
Control	28.8 (4.8) 21.2–37.5 (28.1)	36.5 (14.4) 19.8–68.8 (32.3)	17.1 (3.2) 11.2–24.0 (16.4)	52331 (9344) 34573–73445 (50965)	30931 (8735) 19576–50238 (29600)
Sarcoidosis	19.2 (5.4) 7.4–37.5 (19.4)	25.5 (11.8) 5.8–52.6 (25.0)	12.7 (2.9) 6.0–19.9 (12.5)	46890 (10737) 22247–74559 (45975)	19243 (5407) 8657–32188 (18921)

^a^Mean (standard deviation).

^b^Range (median).

**Figure 3:**
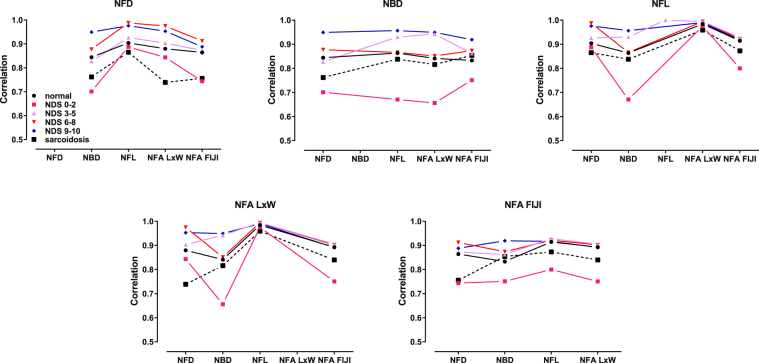
Interclass correlation coefficients of the CCM variables assessed as a function of neuropathic disability. The continuous variables NFL, NFA WxL, and NFA FIJI are generally characterized by having the tightest correlation with other measures. In contrast, the discrete variables NFD and NBD are less well correlated. [NDS = Neuropathic Disability Score. Points are connected as a visual aid].

**Figure 4:**
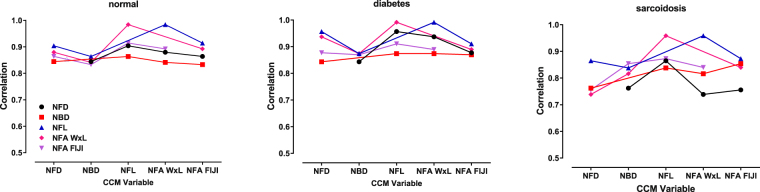
Interclass correlation coefficients of CCM variables as a function of subject group. Notably, the correlation between CCM variables in the healthy control group were highest, the sarcoidosis group lowest, with the diabetes group intermediate. [Points are connected as a visual aid].

The NDS 0–2 and sarcoidosis groups were also characterized by having the lowest interclass correlation between CCM variables, irrespective of the variable assessed, compared to diabetic patients with mild and severe neuropathy (Fig. 3). Additionally, the interclass correlation coefficients between the different CCM variables were calculated for the controls, diabetes, and sarcoidosis study groups as summarized in Fig. 4. NFD, NFL, NBD, NFA WxL, and NFA FIJI exhibited similar high correlations with each other, with Pearson correlation coefficients ranging from 0.83–0.91 for the control group, 0.84–0.96 for the diabetes group, and 0.75–0.87 for the sarcoidosis group. Despite the diabetes group representing a full range of neuropathic disability, correlation between CCM variables was higher than it was for the patients with sarcoidosis. Therefore, within these two patient groups characterized by significant small nerve fiber loss, the actual mean values determined by CCM analysis varied as a function of variable assessed. Not surprisingly, NFL and NFA WxL exhibited the highest correlations, as NFL is used to calculate NFA WxL.

**Figure 5:**
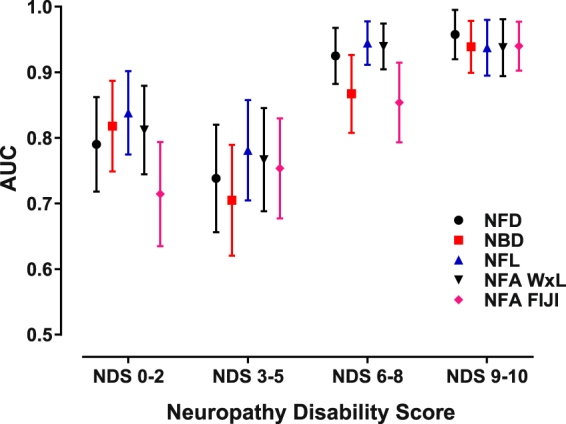
All CCM variables performed similarly in discriminating patients having different degrees of neuropathic disability from normal subjects. The area under the curve (AUC) of the receiver operating characteristic for each variable was significant and similar for each level of neuropathic severity. See text for sensitivity/specificity and cut point data. [Error bars: SEM].

The area under the receiver operating characteristics curves (AUC) obtained by logistic regression summarize the ability of each CCM variable to discriminate diabetic patients with neuropathy from healthy control subjects (Fig. 5). The discrimination between control subjects and patients with no or mild neuropathy were in the 0.7–0.8 AUC range, whereas patients with more severe disability exhibited significantly higher AUCs, approaching 0.95 for the most severely affected group. Surprisingly, the NDS 3–5 disability group had lower AUCs for all CCM variables, as compared to the NDS 0–2 group in spite of the fact that the two groups had similar mean CCM variables. Overall, the results show that the mean AUCs corresponding to each variable successfully discriminate patients with moderate to severe neuropathy from healthy control subjects (p values all < 0.01, except for NFA FIJI (NDS 0–2; p = 0.019 and NBD (NDS 3–5; p = 0.025) with respect to an AUC of 0.5, i.e., no discrimination), with no significant statistical differences observed between the different CCM analysis variables.

Cut off points for discrimination of patients having neuropathy from the normal controls were also calculated from the ROC curves by maximizing the number of true positives and true negatives. For the diabetes patients considered as a group, the cut point for NFD was 23.4 fibers/mm^2^ (i.e., values < 23.4 fibers/mm^2^ optimally distinguished between this group from control), with a sensitivity of 90% and specificity of 69%. The cut point for NBD was 23.4 branches/mm^2^ (sensitivity = 86%; specificity = 71%), for NFL was 12.3 mm/mm^2^ (sensitivity = 96%; specificity = 68%), and for NFA FIJI was 19,128 µm^2^/mm^2^ (sensitivity = 94%; specificity = 59%). The cut points calculated from CCM data obtained from the sarcoidosis patients was similar: NFD ≤ 23.8 fibers/mm^2^ (sensitivity 84%; specificity = 88%), NBD ≤ 26.0 branches/mm^2^ (sensitivity 60%; specificity = 74%), NFL ≤ 14.4 mm/mm^2^ (sensitivity 84%; specificity = 73%), and NFA FIJI ≤ 25,060 µm/mm^2^ (sensitivity = 89%; specificity = 74%).

### Nerve Fiber Width Distribution

**Figure 6:**
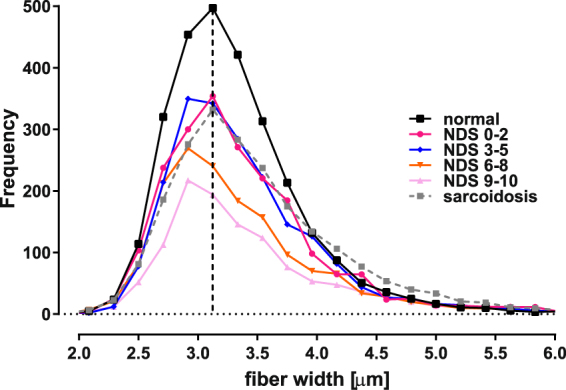
The nerve fiber bundle width frequency distribution illustrates that a progressive dropout of nerve fiber bundles surrounding the mean of normal controls (3.13 µm; dashed vertical line) occurs as a function of increasing severity of neuropathic disability. The relative abundance of nerve fiber bundles having widths in the 2.7–4.0 µm range preferentially decreased compared to smaller and larger ones, resulting in asymmetrical curves for the neuropathy groups. Note that although the sarcoidosis group distribution was similar to the milder diabetic neuropathy groups in the <4.0 µm range, there was relative increase in the abundance of wider fibers compared to the diabetes subjects. (Also refer to Fig. S2).

Typical CCM images of subjects with severe neuropathy exhibit a loss of small nerve fibers when compared to healthy controls, with the remaining fibers appearing to be relatively thick (Fig. 1). The normalized width frequency distributions for individual subjects varied such that those having the most severe neuropathy were characterized by width frequency distributions which varied widely with respect to amplitude and half-width maximum (Fig S1). The average frequency width distribution of nerve fiber bundles of normal subjects was that of a nearly symmetrical curve having a maximum centered on a fiber width of ~3.2 µm (dashed vertical line in Fig. 6). The width frequency distributions can be fitted equivalently using either a Gaussian or log normal model. In contrast, the width frequency distribution of subjects was progressively reduced in the ~2.8–4.0 µm range with increasing neuropathic severity, to about 30–50% of normal (Fig. S2). Both very thin ( < ~2.8 µm) and thick ( > ~4.0 µm) nerve fibers were relatively preserved with a resultant shift in the width frequency distribution curve towards larger nerve fiber bundle diameters with increasing severity of neuropathy. Notably, the sarcoidosis group exhibited a more striking enrichment of thicker nerve fiber bundles, compared to the other groups (Figs 6 and S2). This accounts for the observation that the NFA WxL values for the sarcoidosis group approximated the controls (Fig. 2D,F) rather than any of the groups with diabetes. The average nerve fiber bundle width obtained from the entire CCM image increased in a strong inverse relationship for each CCM variable (Fig. S3).

### The relationship between NFL and NFA is nonlinear

**Figure 7:**
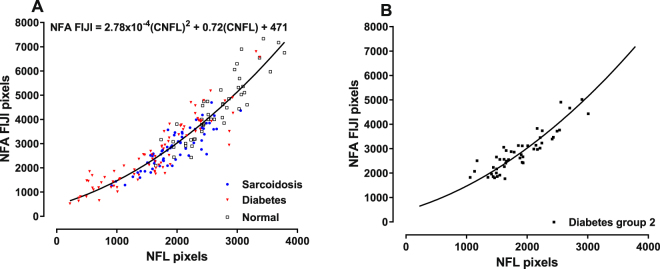
(**A**) The relationship between NFL and NFA FIJI is characterized by a quadratic function. The change in slope of this curve shows that NFA changes more rapidly than NFL as nerve fiber densities approach normal (right portion of the curve). Conversely, differences bewteen these two variables are more similar in cases of severe neuropathy (left portion of the curve). (**B**) CCM data obtained using an independent (cross-validation) group of Type 2 diabetic subjects fall on the prediction curve from panel A.

The subject groups were further analyzed to determine the relationship between NFL and NFA FIJI, two variables calculated using the number of pixels contained within the identified image targets. A plot of NFL versus NFA FIJI pixel values obtained by analyzing the same images from 192 individuals (Fig. 7A) shows that data obtained from the diabetic subjects with severe neuropathy is distributed within the left (lower) portion of the curve, while the sarcoid data set is restricted to the central portion of the curve, and control subjects within the right (upper) portion. Clearly the relationship is nonlinear as the slope of the curve becomes progressively steeper as corneal nerve fiber abundance increases. Linear regression analysis performed using the Passing-Bablok technique showed that the highly significant relationship between these two variables is given by the quadratic equation NFA FIJI = 2.78 × 10^−4^ (NFL)^2^ + 0.72(NFL) + 471 (solid curve in Fig. 7; p < 0.0001). This function predicts that differences between two nearby points on the region of the curve corresponding to more abundant nerve fibers will be larger for NFA FIJI than for NFL. For example, if a subset of subjects is established from the data set illustrated in Fig. 7A by arbitrarily selecting subjects with NFL pixels ≥ 1500 (i.e., ≥ 9.8 mm/mm^2^), groups of 50 subjects with diabetes and 55 with sarcoidosis result, having an overlapping distribution. These groups are characterized by having a mean NFL (and SEM) of 13.79 (0.38) mm/mm^2^ for diabetes and 13.35 (0.37) for sarcoidosis (a difference of ~3.2% of the mean value for the entire group), which is not statistically different (p = 0.409). In contrast, the corresponding mean NFA FIJI values are 23,238 (904) µm^2^/mm^2^ for diabetes compared to 20,460 (862) µm^2^/mm^2^ for sarcoidosis (a difference of ~12.7% of the mean value for the entire group), which is significantly different (p = 0.028). Alternatively, logistic regression performed using the same data set shows that these groups cannot be differentiated using NFL, in contrast to NFA FIJI which does provide significant discrimination. Finally, NFL and NFA FIJI data obtained from an independent diabetes group (diabetes group 2; n = 48) also fit this regression curve well (r^2^ = 0.80; Fig. 7B), providing cross-validation confirmation for this relationship.

### Change in CCM variables following intervention with cibinetide

**Figure 8:**
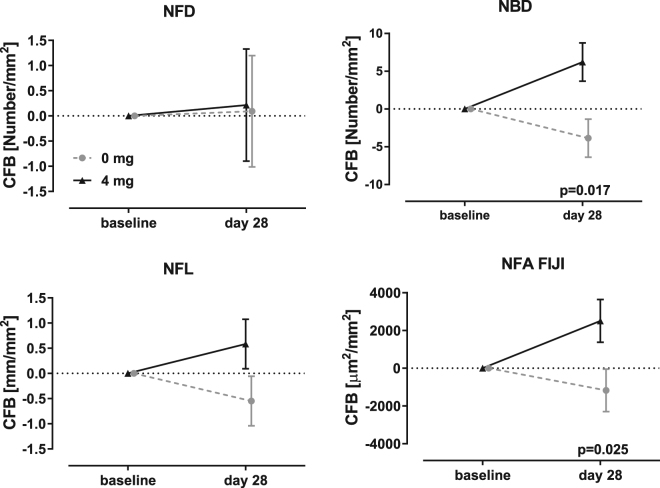
The variables NBD and NFA FIJI show significant changes in nerve fiber morphology following 28 days in the cibinetide treatment arm(black triangles, solid line), but not for placebo (grey circles, dashed line). In contrast, assessments using the variables NFD and NFL do not show a significant change. (Error bars indicate SEM).

Based on the descriptive statistics of the sarcoidosis data set, the minimum detectable change (MDC) was NFD = 1.91 fibers/mm^2^; NBD = 4.19 branches/mm^2^; NFL = 1.03 mm/mm^2^ and NFA FIJI = 1922 µm^2^/mm^2^. The sarcoidosis data set was evaluated at baseline and following 28 days of daily administration of 4 mg of cibinetide or placebo. NFD showed no change (Fig. 8). The change in NFL was −0.57 ± 0.50 mm/mm^2^ for placebo and 0.56 ± 0.50 mm/mm^2^ for 4 mg cibinetide, giving a net change of 1.15, approximating the MDC, but ANCOVA did not show a significant difference between the groups. In comparison, the change in NFA FIJI was above the MDC being −1196 ± 1142 µm^2^/mm^2^ for placebo and 2521 ± 1142 µm^2^/mm^2^ with a net change of 3717 for the 4 mg dosage group, which was significantly different. A corresponding increase in the abundance of small nerve fiber widths in the 2.8–4.0 µm range was observed for the 4 mg cibinetide group, compared to placebo (Fig. S4). NBD also showed a significant difference between the placebo and active groups following cibinetide administration.

## Discussion

These data confirm that CCM using automated image analysis is an excellent methodology for quantification of corneal nerve morphology and provides a reliable estimate of small nerve fiber abundance. All CCM variables evaluated demonstrated equivalent ability to discriminate patients with diabetic neuropathy from healthy controls, based on comparable AUCs of the receiver operating curves, a finding which is similar to the results of a previous study using ACCmetrics which observed AUCs within the ~0.75 range^15^. Additionally, the cut off points used to distinguish neuropathy patients with diabetes from normal controls are similar to those determined by Petropoulos *et al*.^7^ for subjects with diabetic neuropathy established using nerve conduction velocity, Neuropathy Disability Score, and vibratory-thermal sensory thresholds to diagnose the presence of neuropathy.

Moreover, these results demonstrate that additional useful information is contained within each CCM image in the form of the width distribution of nerve fiber bundles and total nerve fiber area, which also vary as a function of neuropathic severity. Therefore, in addition to a reduction in nerve fiber length, density and branch density, the relative loss of nerve fiber bundles in the 2.8–4.0 µm range appears to be an additional metric to characterize small fiber neuropathy. Thus, with increasing corneal nerve fiber loss, a preferential drop out of nerve fiber bundles around the mean width was observed, shifting the width frequency distribution towards thicker fiber bundles. It is interesting that the relative proportion of the very small fibers (<2.7 µm) is not reduced as much as fibers in the midrange of width as shown by the non-symmetrical curves around the normal mean (Fig. 6). It is possible that the fiber bundles of smaller widths which appear to be relatively preserved represent a thinning process arising from the time-dependent drop out of individual nerve fibers from wider bundles. Alternatively, or in addition to nerve fiber drop out, the enrichment of wider fiber bundles could occur via individual fiber swelling, which is a known characteristic of small fiber neuropathy^21^. Additional longitudinal study and electron microscopy level histology correlated with the CCM findings will be required to investigate these possibilities further.

The data also suggest that the differences in the caliber of nerve fiber bundles may allow CCM methodology to identify specific types of neuropathy. In this regard, the comparison between diabetes- and sarcoidosis-associated SFN is interesting. In the present study, NFD, NBD, and NFL of the sarcoidosis subjects overlapped with the NDS (0–2) and NDS (3–5) diabetes groups. However, although both groups had a similar proportional reduction in nerve fibers around the normal mean (i.e., in the 2.7–4.0 µm range), a large relative increase in wider nerve fiber bundles was noted in the sarcoidosis group (Figs 6 and S2). This characteristic of the sarcoidosis group contributed to the poorer interclass correlation coefficients noted when compared to the diabetes group. Further studies are required to establish whether both thin and thick corneal nerve fiber bundles drop out to a more balanced extent in subjects with mild diabetic neuropathy compared to subjects having sarcoidosis, or whether sarcoid neuropathy is characterized by a preponderance of smaller nerve fiber bundle loss. Additionally, it will be interesting to determine whether nerve fiber bundle width distributions in SFN of other etiologies are different or similar to the two neuropathy groups studied here.

Notably, the relationship between NFL and NFA FIJI in control subjects or patients with neuropathy fall along the same regression line, indicating that the morphological characteristics of the corneal nerve fiber plexus are predictably related to nerve fiber abundance, regardless of the presence or absence of neuropathy or disease severity. Although the individual CCM variables were highly intercorrelated, the values corresponding to subjects with milder neuropathy were lower than those for subjects with severe neuropathy. This finding is consistent with a variable impact of the disease process on different morphological components of the nerve fiber plexus, but also may reflect the relatively crude assessment of neuropathic deficits using NDS, especially for milder neuropathy. Therefore, although each CCM variable is equally useful determine the existence of severe neuropathy, NFL and NFA appear to be more sensitive discriminators of the presence of mild neuropathy. In this respect, however, it is important to note that NFL is a one-dimensional variable (length), whereas NFA is two-dimensional (length times width). Therefore, NFA provides additional information, especially for increasing complexity of corneal nerve plexus, i.e., for states of mild neuropathy. These data are supportive of the conclusion that NFA FIJI may have particular utility for identifying early nerve fiber degeneration.

It is notable that diabetic subjects without apparent neuropathy (NDS 0–2) exhibited a much wider range of values for the CCM variables examined compared to those having mild neuropathy. This observation is presumably explained the inclusion of individuals deemed not to have neuropathy based on the clinical observation of preserved large fiber-mediated deep tendon reflexes and touch and vibration sensitivity which are components of the NDS, but who nevertheless have SNF loss when assessed using CCM. This possibility is underscored by the results of longitudinal studies which show that corneal small nerve fiber loss is an prominent early feature of neuropathy e.g. subjects with impaired glucose tolerance demonstrate a loss of small nerve fibers before abnormalities are noted in electrophysiological studies^30^.

In relation to surrogate end points for clinical trials assessing therapeutic benefit, both NBD and NFA FIJI show a significant change in the corneal nerve fiber morphology after treatment with cibinetide. This is particularly significant as 28 days is a short time frame to assess potential changes in the corneal nerve plexus, even though rapid fiber changes ranging from 5–26 µm/week have been observed in the normal cornea^31^. We have previously independently confirmed that SNF abundance increases following cibinetide administration in this study by documenting a significant increase of growth-associated protein-43 immunoreactive fibers in concurrently-obtained skin biopsies^27^. The reason why NFA FIJI is a sensitive assessment of change in the underlying small nerve fiber plexus, while change in NFD, NFL or NFA WxL are inadequate measures, can potentially be explained by several factors. First, NFD is a discrete variable and therefore changes that fall between integers are rounded up or down to a whole number and therefore NFD will vary only if relatively large changes in the nerve fiber plexus occur. In contrast to NFD, NFL and NFA approximate continuous distributions as these are based on image pixels, which are much better suited to detect small changes within the nerve fiber plexus. Second, NFL as determined by ACCmetrics is derived using highly processed images which may not fully represent the fine structure of the original image. It will be important to examine the relationship between manually determined NFL and NFA FIJI to determine how important a factor image processing may be. Finally, NFA WxL is a derived variable, based on highly processed images and since NFL is used for the NFA WxL calculation, these variables are highly correlated and have similar discriminatory properties.

There are several limitations of this study. First, this is a post hoc analysis using groups of relatively small sample sizes. Larger studies over a wide range of neuropathic severity, determined using independent diagnostic modalities such as neurophysiology or IENFD, will be required to confirm and extend these results. Also, although subjects from two different etiologies for SFN were studied, additional cases of small fiber neuropathies of presumed inflammatory or non-inflammatory etiology should be evaluated to determine the generalizability of these results.

In conclusion, the corneal nerve fiber bundle size distribution provides additional CCM-derived variables to identify and quantify the severity of SFN loss. Although automated NFD, NBD, NFL, and NFA FIJI all appear to be useful for identifying patients having neuropathy, NFA FIJI has the widest dynamic range, and is especially useful for assessing patients having mild to moderate neuropathy. Furthermore, NFA-FIJI as a measure of nerve fiber morphology is sensitive enough to detect small improvements in the corneal nerve fiber plexus over short periods of time, and therefore may provide a window of opportunity to identify potentially beneficial therapies for SFN.

## Methods

### Subjects

CCM data were obtained from a group of healthy control subjects and compared to subjects with diabetes. Two additional data sets collected in previous clinical trials (see below) were also utilized. These studies were performed in accordance with the Declaration of Helsinki and each subject provided informed consent before evaluation.

48 control subjects and 81 subjects with diabetes (diabetes group 1) were recruited after receiving ethical approval (University of Manchester, Manchester, UK). The severity of neuropathy was classified using the Neuropathy Disability Score (NDS) to stratify diabetic subjects with no (NDS-0–2), mild (NDS-3–5), moderate (NDS-6–8), or severe (NDS-9–10) neuropathy^32^. An additional group of patients with Type 2 diabetes with small fiber neuropathy was used for cross-validation (diabetes group 2; n = 48). This study was registered (Netherlands Clinical Trials Registry: NTR3858) and the results have been published^26^.

For assessment of longitudinal intervention, subjects with sarcoidosis and symptoms of painful SFN, were also evaluated following ethical and subject’s approval (Cleveland Clinic, Cleveland, OH and Leiden University Medical Center, NL; EudraCT (2013-003016-45); Clinicaltrials.gov (NCT02039687) at baseline (n = 63) and at day 28 following daily self-administration of placebo (n = 16) or 4 mg (n = 15) cibinetide subcutaneously (Table 1). Based on preclinical and prior clinical data, it was expected that the 4 mg dose would provide drug exposure sufficient to support nerve fiber regrowth. Additional aspects and results of this trial have been reported previously^27^. The sarcoidosis group was characterized by having moderate pain severity at baseline (mean score of 6.0 (range 3.3–9.8) using the Brief Pain Inventory severity scale which ranges from 0 to 10) and mean IENFD density of 5.5 fibers/mm (range 0.3–13.0) in distal lower limb biopsies. Using an IENFD cut point of 8.8 fibers/mm as determined by Vlckova-Moravcova *et al*.^33^, 56 subjects (89% of total) would be classified as having SFN based on cutaneous nerve fiber density.

### Corneal Confocal Microscopy and Image Analysis

Corneal confocal images of the sub-basal nerve layer of the central cornea were acquired using the Rostock Corneal Module of the Heidelberg Retinal Tomograph III (HRT III) using a previously established protocol^34^. For the diagnostic group (diabetes versus healthy control subjects), 5–8 images (median 6) of corneal nerves from the sub-basal layer of one eye were selected on the basis of technical quality by an expert (MF) blinded as to subject identity. 579 images were selected and analyzed and the results averaged for each individual. For the longitudinal assessment group (sarcoidosis patients), as the SFNL associated with this disease has been reported to be “patchy” in distribution^35^, both eyes were imaged and 2479 images were selected based on image quality by an expert (MB) blind to treatment group (6–18; median of 10 per eye corresponding to baseline and the 28 day follow up period). Data obtained from the images from each eye were averaged and the mean of both eyes used for analyses.

The image data obtained by the HRT III are derived using an intentionally very limited axial resolution (depth of focus) of 7.6 µm^36^. The sub-basal nerve plexus which contains the nerve bundles consists of a single, very thin (<10 µm) layer parallel to the corneal surface^37^. Therefore, each technically acceptable image likely samples the entire sub-basal layer. To reduce the unlikely possibility that images could be acquired at slightly different corneal depths within the sub-basal plexus, a standard protocol for acquiring images was used, and the results of multiple independent images averaged.

Automated data analysis was performed using two different algorithms: ACCmetrics and a custom-developed macro for FIJI. ACCmetrics^15^ provides individual estimates of corneal nerve fiber density, corneal nerve branch density and corneal nerve fiber length. Nerve fiber width distribution is also generated as the frequency of occurrence of specific fiber widths (ranging from 1 to a maximum of 8 pixels) determined perpendicularly to each pixel constituting the long axis of identified nerve fibers^15^. NFA was calculated as the total number of width pixels multiplied by the total number of length pixels (NFA WxL).

Although the ACCmetrics algorithm improves target identification, the image processing steps utilized degrade and de-emphasize fine structural details such as nerve fiber beading and subtle changes in nerve fiber width, and additionally bridge small gaps along the length of each nerve fiber. Therefore, a second algorithm was developed to determine NFA. This method employs a custom-developed macro for FIJI (NFA FIJI), a public-domain image analysis program, version 1.47e. The program creates a binary image of the corneal nerve fibers by applying a Gaussian filter to enhance target contrast and adjusts detection threshold based on background brightness, followed by use of the eigenvalues of the Hessian matrix to identify tubular nerve fiber structures^38^. NFA FIJI is defined as the image area (µm^2^) covered by the resulting mask, and is expressed as per mm^2^ of corneal surface area.

### Statistical Analysis

Normality of the data sets for each CCM variable was assessed using the Shapiro-Wilks W test. NFA FIJI data were found to differ significantly from a normal distribution and were converted into a normal distribution for analysis by square root transformation. Comparisons between group means was accomplished using interclass correlation analysis and Tukey’s multiple comparison test. These normalized data were also used to determine the relationship between NFL and NFA in pixel units, employing linear regression analysis as per the methodology of Passing and Blalok. This analytical method allows for the direct comparison of measurements obtained using different methodologies and which exhibit different variances^39^. The resulting linear regression equation was then applied to CCM data obtained from 48 additional type 2 diabetic patients^26^ (diabetes group 2) with neuropathic pain consistent with SNF loss. The baseline characteristics of Diabetes Group 2 were notable for reporting an average pain level over the last month of 5.7 (range: 1–10) as determined by the PainDetect instrument^40^ which uses a scale from 0–10. Additionally, the mean IENFD at baseline was 5.2 fibers/mm (range: 0–26.5).

Logistic regression was carried out for each NDS-based severity group of patients with diabetes versus control subjects and receiver operator curves constructed. Comparison of the results of different analysis algorithms performed upon the same data set was accomplished by comparing the area under the ROCs using the methodology of Hanley and McNeil^41^. This technique accounts for the significant correlation between comparisons of individual data points obtained from the same image and is analogous to the paired t-test. Cut points for optimum discrimination between the neuropathy groups and normal controls were estimated by determining the point on the ROC which corresponded to the maximum number of true positives and true negatives. Longitudinal changes in CCM variables in the sarcoidosis neuropathy group were observed to depend upon the baseline values. Therefore, to determine change after 28 days of cibinetide or placebo administration, analysis of covariance was carried out using treatment as a factor and with baseline value as a covariate. For change in CCM variables, the minimum detectable difference was calculated as the SEM × 1.96 × $\sqrt{2}$^42^. The statistical software employed was JMP (SAS, Cary, NC; version 11).

### Data availability

The datasets generated and analyzed in the current study are available from the corresponding author on reasonable request.

## Electronic supplementary material

Supplementary Information
