# Supplementary material for: Corneal nerve fiber size adds utility to the diagnosis and assessment of therapeutic response in patients with small fiber neuropathy
Source: Sci Rep. 2018 Mar 16;8:4734. doi: 10.1038/s41598-018-23107-w (PMC5856845; doi:10.1038/s41598-018-23107-w)

Supplementary material.

Corneal nerve fiber size adds utility to the diagnosis and assessment of therapeutic response in patients with small fiber neuropathy

Michael Brines, Daniel A. Culver, Maryam Ferdousi, Martijn R Tannemaat, Monique van Velzen, Albert Dahan, Rayaz A. Malik

Figure S1. Individual width frequency distributions as a function of neuropathic disability. The middle dashed line indicates the mean width maximum frequency. Other dashed lines are for reference at 2.5 (thin) and 4.5 (thick) pixels. As neuropathy severity increases, the frequency distributions shift towards thicker fibers. [Dashed lines are provided to facilitate comparisons at 2.5 µm, 3.1 µm (the center of the frequency distribution for healthy controls), and 4.5 µm.]

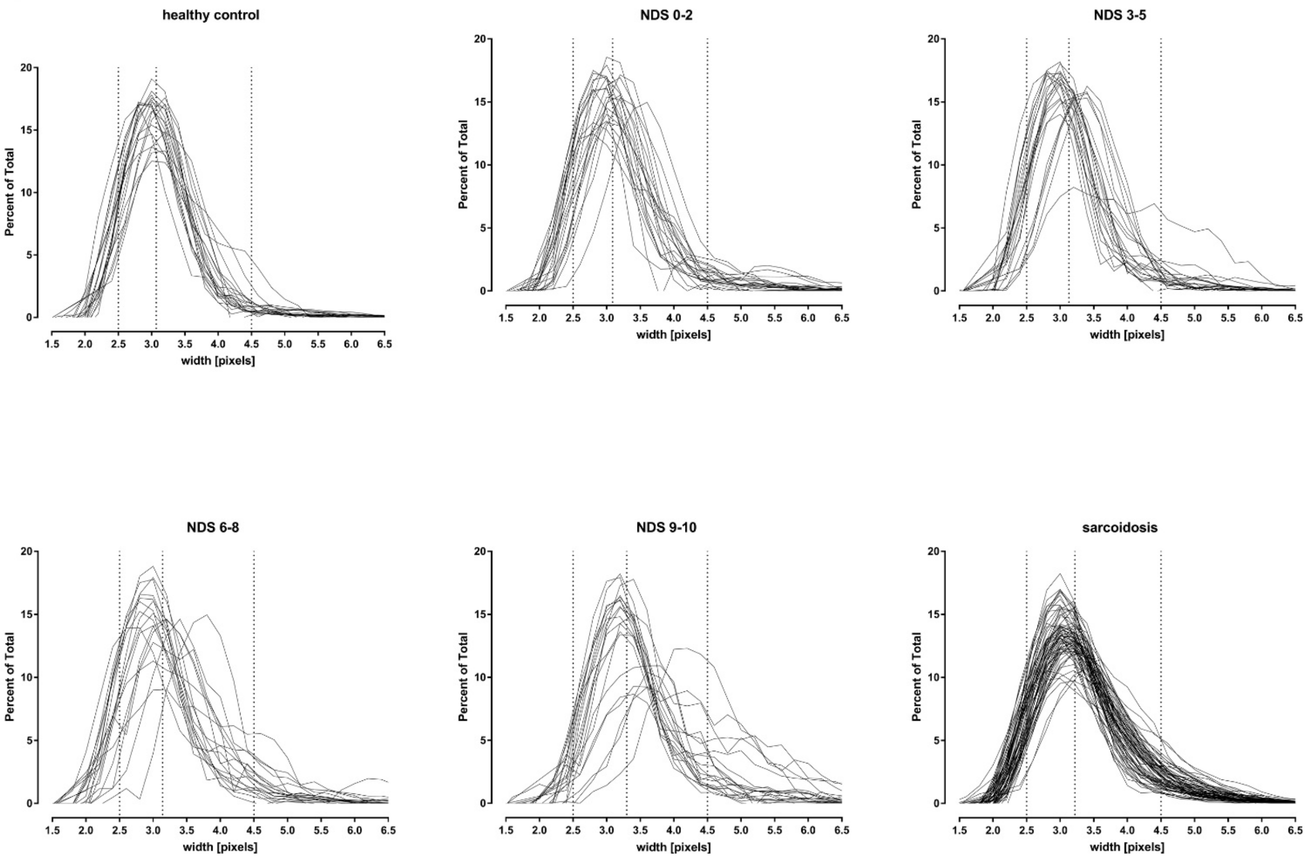

Figure S2. Normalized frequency width distributions show that a reduction of the thinner nerve fiber bundles occurs for increasing neuropathic severity compared to the mean width of normal subjects, leading to a relative increase in the proportion of wider fibers. Note that although the sarcoidosis group and NDS (0-2) and NDS (3-5) had similar reductions in the midrange of nerve fiber widths, the sarcoidosis group was enriched in nerve fiber bundles wider than 4.0 µm.

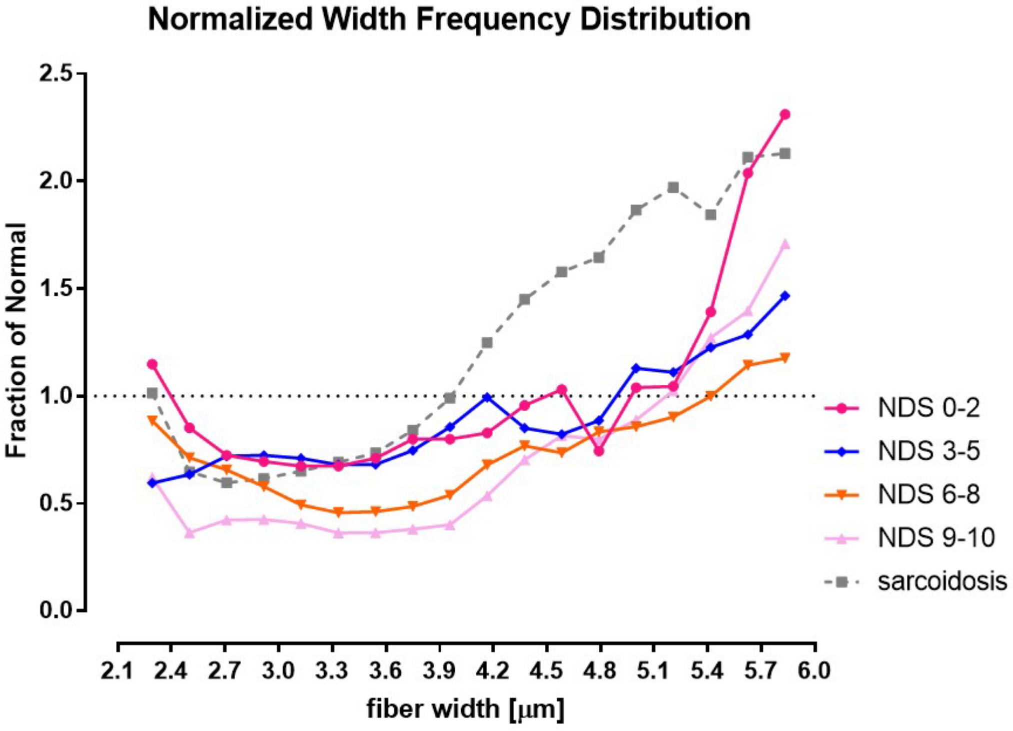

Figure S3. A strong inverse relationship exists between CCM variables and the mean nerve fiber width as a function of increasing neuropathic disability.

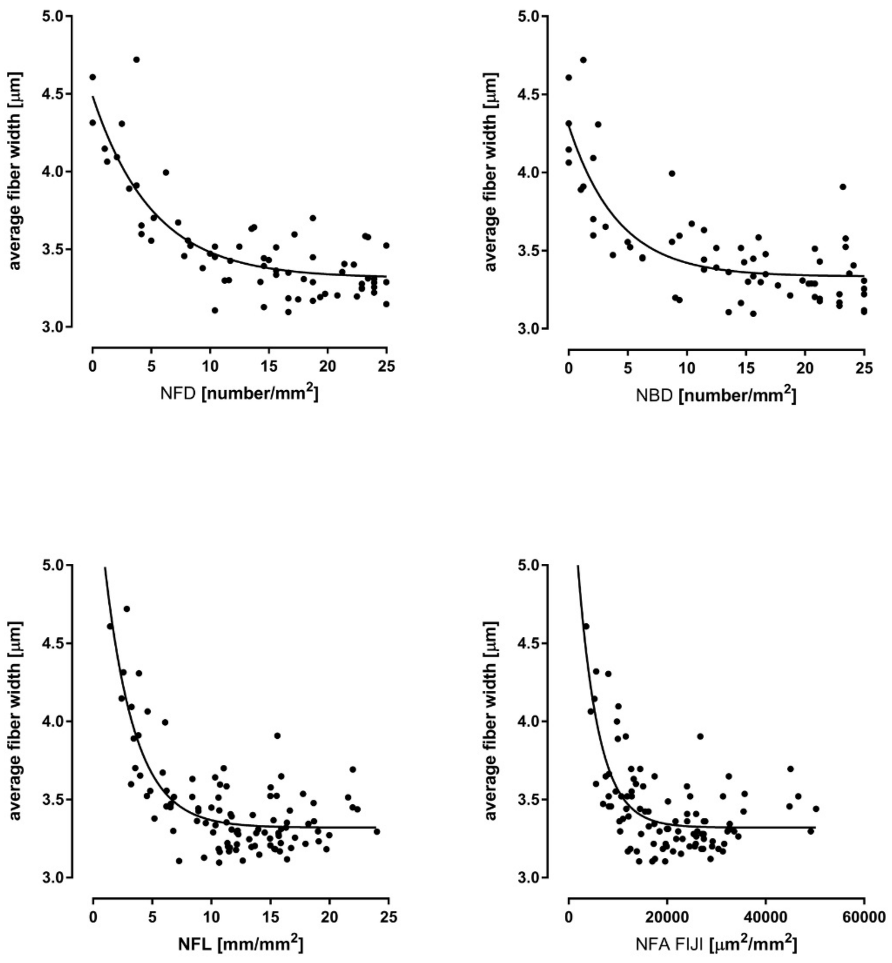

Figure S4. After 28 days of treatment, midrange (2.7-4.0 µm) nerve fiber bundles increased in relative abundance for subjects within the active drug arm. (Error bars: SEM)

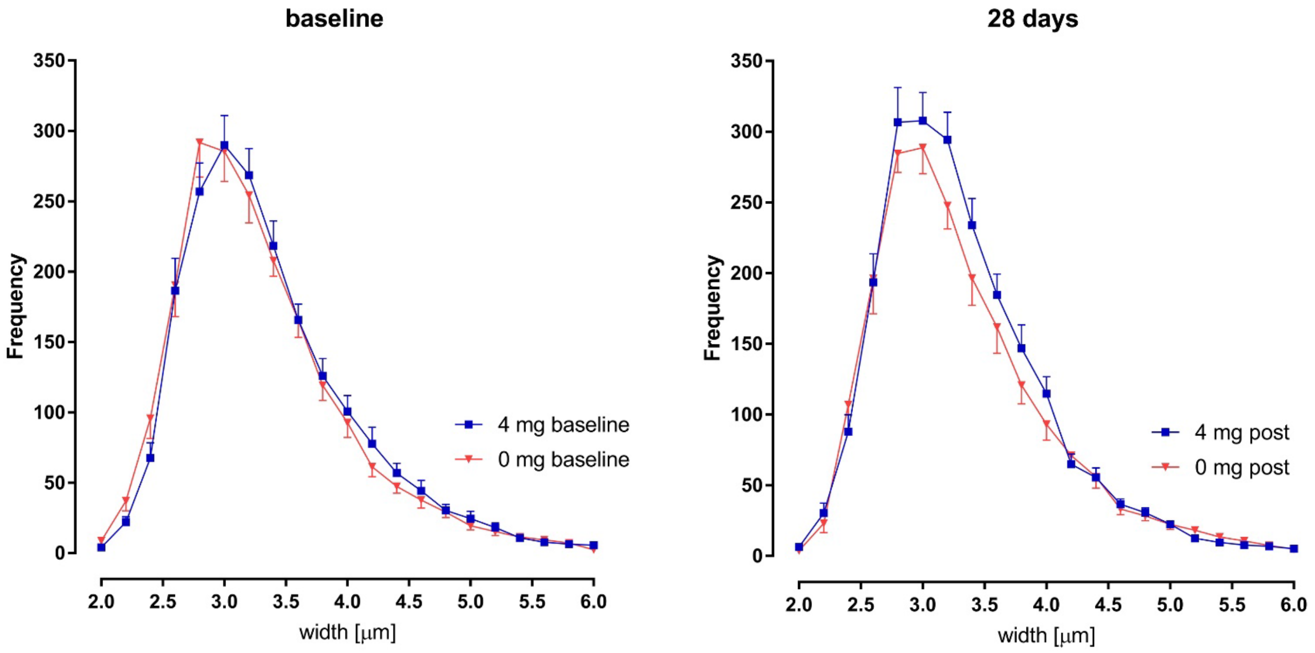

Supplement: Supplementary file 1 — Supplementary Information [file 41598_2018_23107_MOESM1_ESM.pdf]
